# Continuous Hemofiltration During Extracorporeal Membrane Oxygenation in Adult Septic Shock: A Comparative Cohort Analysis

**DOI:** 10.3390/biomedicines13081829

**Published:** 2025-07-26

**Authors:** Nicoleta Barbura, Tamara Mirela Porosnicu, Marius Papurica, Mihail-Alexandru Badea, Ovidiu Bedreag, Felix Bratosin, Voichita Elena Lazureanu

**Affiliations:** 1 Doctoral School, “Victor Babes” University of Medicine and Pharmacy, Eftimie Murgu Square 2, 300041 Timisoara, Romania; nicoleta.barbura@umft.ro; 2 Anaesthesia and Intensive Care Research Center, Faculty of Medicine, “Victor Babes” University of Medicine and Pharmacy, Eftimie Murgu Square 2, 300041 Timisoara, Romania; bedreag.ovidiu@umft.ro; 3 Dermatology Department, The George Emil Palade University of Medicine, Pharmacy, Science, and Technology, 540139 Targu Mureș, Romania; mihail.badea@umfst.ro; 4 Department of Infectious Disease, “Victor Babes” University of Medicine and Pharmacy, Eftimie Murgu Square 2, 300041 Timisoara, Romania; felix.bratosin@umft.ro (F.B.); lazureanu.voichita@umft.ro (V.E.L.)

**Keywords:** septic shock, extracorporeal membrane oxygenation, hemofiltration, renal replacement therapy, critical care outcomes

## Abstract

**Background and Objectives***:* Severe sepsis complicated by refractory shock is associated with high mortality. Adding continuous hemofiltration to venovenous extracorporeal membrane oxygenation (ECMO) may accelerate clearance of inflammatory mediators and improve haemodynamic stability, but evidence remains limited. We analysed 44 consecutive septic-shock patients treated with combined ECMO-hemofiltration (ECMO group) and compared them with 92 septic-shock patients managed without ECMO or renal replacement therapy (non-ECMO group). **Methods***:* This retrospective single-centre study reviewed adults admitted between January 2018 and March 2025. Demographic, haemodynamic, laboratory and outcome data were extracted from electronic records. Primary outcome was 28-day mortality; secondary outcomes included intensive-care-unit (ICU) length-of-stay, vasopressor-free days, and change in Sequential Organ Failure Assessment (SOFA) score at 72 h. **Results***:* Baseline age (49.2 ± 15.3 vs. 52.6 ± 16.1 years; *p* = 0.28) and APACHE II (27.8 ± 5.7 vs. 26.9 ± 6.0; *p* = 0.41) were comparable. At 24 h, mean arterial pressure rose from 52.3 ± 7.4 mmHg to 67.8 ± 9.1 mmHg in the ECMO group (mean change [∆] + 15.5 mmHg, *p* < 0.001). Controls exhibited a modest 4.9 mmHg rise that did not reach statistical significance (*p* = 0.07). Inflammatory markers decreased more sharply with ECMO (IL-6 ∆ −778 pg mL^−1^ vs. −248 pg mL^−1^, *p* < 0.001). SOFA fell by 3.6 ± 2.2 points with ECMO versus 1.6 ± 2.4 in controls (*p* = 0.01). Twenty-eight-day mortality did not differ (40.9% vs. 48.9%, *p* = 0.43), but ICU stay was longer with ECMO (median 12.5 vs. 9.3 days, *p* = 0.002). ΔIL-6 correlated with ΔSOFA (ρ = 0.46, *p* = 0.004). **Conclusions***:* ECMO-assisted hemofiltration improved early haemodynamics and organ-failure scores and accelerated cytokine clearance, although crude mortality remained unchanged. Larger prospective trials are warranted to clarify survival benefit and optimal patient selection.

## 1. Introduction

Sepsis remains a formidable global health threat, accounting for an estimated 48.9 million incident cases and 11 million deaths in 2017 alone [1]. Contemporary Surviving Sepsis Campaign (SSC) guidelines emphasise rapid recognition, bundled resuscitation and early vasopressor support, yet worldwide adherence is uneven and case-fatality for septic shock continues to hover between one in three and one in six patients [2]. Among those requiring catecholamine doses > 0.5 µg kg^−1^ min^−1^, 28-day mortality routinely exceeds 50 percent [3], underscoring the need for adjunctive strategies capable of modulating the dysregulated host response rather than merely supporting macrocirculation.

Debate persists regarding the optimal timing of norepinephrine. A 2025 meta-analysis, including 6661 patients, found that administration within the first three hours after shock recognition conferred a survival advantage, whereas “extremely early” (<1 h) initiation showed no benefit and, in some subgroups, potential harm [4]. Mechanical support has therefore been explored as a rescue option once conventional vasopressor titration stalls. A 2021 individual-participant-data meta-analysis of 1461 adults treated with veno-arterial extracorporeal membrane oxygenation (ECMO) for refractory septic shock reported an overall survival of 36 percent, rising to 52 percent when left-ventricular ejection fraction was <30 percent at cannulation [5], suggesting that extracorporeal flow may offset sepsis-induced cardiomyopathy in carefully selected patients.

Acute kidney injury (AKI) complicates up to 70 percent of ECMO runs and independently doubles the odds of death [6]. Coupling continuous renal replacement therapy (CRRT) to the ECMO circuit offers logistical simplicity—shared access, unified anticoagulation and stable ultrafiltration—but raises questions about membrane performance and haemodynamic drag. A 2014 systematic review reported a pooled mortality of 51 percent for combined ECMO-CRRT, identical to ECMO alone, but highlighted markedly lower vasopressor requirements when filtration started within six hours of cannulation [7]. More recently, a propensity-matched study comparing in-line versus parallel CRRT connection demonstrated superior filter life and 24-h cytokine clearance in the integrated configuration without additional circuit thrombosis [8].

High-volume haemofiltration (HVHF) was initially championed for “peak concentration hypothesis” control of circulating mediators. The multicentre IVOIRE trial randomised 140 septic-AKI patients to 70 mL kg^−1^ h^−1^ or 35 mL kg^−1^ h^−1^ and documented faster norepinephrine withdrawal but no mortality benefit [9]. A 2014 systematic review nevertheless confirmed significant reductions in interleukin-6 (IL-6) and vasopressor dose across 25 studies [10], findings echoed in a pilot RCT where 65 mL kg^−1^ h^−1^ HVHF halved catecholamine needs within 24 h [11]. Parallel advances in cytokine-adsorbing hemofilters—particularly polymethyl-methacrylate (PMMA) and AN69-ST—have shown enhanced adsorption of mid-weight molecules such as IL-6 and procalcitonin while preserving antibiotic sieving coefficients [12].

Integrating these modalities into ECMO circuits remains technically challenging. In a 2023 registry analysis, studies have demonstrated that continuous in-line CRRT maintained net ultrafiltration targets without increasing oxygenator failure over a median 152-h run time [13]. Adsorptive cartridges have further reduced vasopressor index by 30–50 percent within 24 h in small case-series [14], and a 2024 systematic review confirmed a significant pooled mean arterial-pressure gain of 9 mmHg with adjunctive hemoadsorption [15]. A retrospective cohort of 76 septic-shock patients treated with oXiris-CRRT reported lower 72-h lactate and a trend toward improved 28-day survival versus matched controls [16].

Despite these encouraging signals, translational gaps persist. Large-animal studies reveal membrane-specific differences in IL-6 adsorption kinetics [17], and broad reviews caution that fluid balance, nutritional depletion, and antimicrobial clearance must be meticulously managed when high-dose filtration or sorbent therapies are combined with extracorporeal oxygenation [18]. Experimental work with polyester-polymer-alloy (PEPA) prototypes shows two-fold greater IL-6 removal than cellulose triacetate in rat sepsis models, providing a platform for next-generation filters [19]. Meanwhile, early short-term (4 h) isovolaemic HVHF administered before refractory hypotension has demonstrated rapid catecholamine sparing in uncontrolled human series [20].

Building on our 2023 institutional protocol mandating concurrent high-flux hemofiltration for septic-shock patients cannulated for venovenoven ECMO when lactate ≥ 4 mmol/L and urine output ≤ 0.3 mL kg^−1^ h^−1^, we undertook a retrospective cohort study to compare haemodynamic trajectories, cytokine kinetics, and clinical outcomes with those of contemporaneous non-ECMO septic-shock cases. We hypothesised that integrated ECMO-HVHF would accelerate physiological stabilisation, expressed as greater improvement in mean arterial pressure and SOFA score at 72 h, relative to conventional critical-care management. 

## 2. Materials and Methods

### 2.1. Study Design & Setting

A single-centre study was conducted between January 2018 and March 2025 at the Victor Babes Hospital of Infectious Diseases, affiliated with the “Victor Babes” University of Medicine and Pharmacy from Timisoara. This observational study was performed in a 24-bed tertiary adult ICU with an established ECMO programme. All consecutive admissions were screened through the electronic clinical information system. Informed consent for personal records being used in future research studies was obtained either from patients on admission or from their legal guardians in case they were incapacitated. 

Two cohorts were formed: (1) ECMO group—patients cannulated for venovenoven ECMO who simultaneously received continuous high-volume hemofiltration; (2) non-ECMO group—septic-shock patients managed with standard resuscitation and organ support without ECMO or CRRT. Ethical approval permitted waiver of informed consent owing to retrospective design.

Eligible patients met Sepsis-3 criteria for septic shock and required norepinephrine ≥0.2 µg kg^−1^ min^−1^ after 30 mL kg^−1^ crystalloid resuscitation. Adults were eligible if they were aged ≥ 18 years, and, for the ECMO cohort, were cannulated within six hours of shock recognition. We excluded pregnancy, intracranial haemorrhage, pre-existing end-stage renal disease, documented treatment limitation orders, and inter-facility transfers already receiving vasopressors for more than twelve hours.

### 2.2. Hemofiltration and ECMO Protocol

ECMO was initiated via femoro-jugular cannulation (23-Fr drainage, 19-Fr return) using centrifugal pumps and polymethylpentene oxygenators. Hemofiltration employed a high-cut-off polyethersulfone filter (surface area 1.8 m^2^) integrated post-oxygenator. Effluent dose targeted 45 mL kg^−1^ h^−1^ with predilution replacement fluid. Regional anticoagulation was maintained with unfractionated heparin aiming for activated partial thromboplastin time 55–70 s. Membrane change occurred every 24 h or earlier if transmembrane pressure exceeded 250 mmHg.

### 2.3. Data Collection

The presumed source of sepsis was pneumonia in 64 patients (47%), abdominal infection in 38 (28%), urinary tract infection in 18 (13%), and soft-tissue or other foci in 16 (12%); distributions did not differ between groups (*p* = 0.81).

Demographic variables (age, sex, body-mass index), comorbidities (obesity, hypertension, chronic obstructive pulmonary disease), and infection source were captured. Physiological and laboratory data were recorded at baseline (pre-ECMO or within 2 h of shock recognition for controls) and at 24 h and 72 h. Parameters included MAP, heart rate, arterial pH, leucocyte count, CRP, PCT, IL-6, lactate dehydrogenase, and ferritin. Organ-failure scores (APACHE II on admission; SOFA daily [21]) and norepinephrine dosage were extracted automatically. Outcomes measured were 28- and 90-day mortality, ICU length-of-stay, ventilator-free and vasopressor-free days to day 28.

### 2.4. Statistical Analysis

Continuous variables found to be normally distributed by the Shapiro–Wilk test are reported as mean ± standard deviation and compared between groups with an independent-samples *t*-test; skewed variables appear as median (inter-quartile range) and are compared with the Mann-Whitney U test. Categorical variables are expressed as n (%) and analysed with χ^2^ or Fisher’s exact tests. Within-group changes from baseline to 24 h were assessed with Wilcoxon signed-rank tests, and the between-group difference-in-difference was examined with unpaired *t*-tests. Correlations were calculated with Spearman’s rho (ρ). All analyses used R 4.3.1; significance was set at *p* < 0.05. Multivariable logistic regression evaluated predictors of 28-day mortality; variables with *p* < 0.10 on univariable analysis entered the model. Binary outcomes were analysed as risk ratios (ECMO + HVHF ÷ standard care) with 95% confidence intervals calculated by the Wald method.

## 3. Results

Mean age differed by only 3.4 years (49.2 years in the ECMO group and 52.6 in the non-ECMO group), and showed a wide overlapping standard deviation, yielding a non-significant *p*-value of 0.28 on independent-samples *t*-test. Sex distribution was virtually identical, with approximately two-thirds male predominance in both groups (68.2% in the ECMO group vs. 68.5% in the non-ECMO group). Importantly, the severity-of-illness metrics (APACHE II and baseline SOFA) were also comparable; APACHE II differed by 0.9 points (*p* = 0.41) and SOFA by 0.4 points (*p* = 0.48), indicating equivalent acute physiological derangement at study entry (Table 1).

Five of the six individual SOFA subscores—cardiovascular, respiratory, renal, coagulation, and central nervous system—declined significantly more in the ECMO group after Bonferroni correction; only the hepatic component did not differ.

Haemodynamic trajectories over the first 24 h underscore the rapid stabilisation afforded by ECMO-assisted hemofiltration. Although baseline MAP was marginally lower in the ECMO group (52.3 mmHg vs. 55.6 mmHg), this small difference reached statistical significance (Student’s *t*), reflecting the clinical decision to escalate support in the sickest subset. Crucially, after 24 h the ECMO cohort achieved a mean MAP of 67.8 mmHg—well above the 65 mmHg guideline target—whereas controls averaged 60.5 mmHg, a between-group divergence of 7.3 mmHg with *p* < 0.001. Heart-rate reduction mirrored this trend: ECMO recipients demonstrated a 26.4 bpm fall versus 10.8 bpm among controls, suggesting better catecholamine tapering and sympathetic quiescence. This is corroborated by norepinephrine requirements, which more than halved in the ECMO group (−0.39 µg kg^−1^ min^−1^) compared with a modest −0.11 µg kg^−1^ min^−1^ decline in the non-ECMO arm (Table 2). 

Analysis of acute-phase and cytokine profiles reveals marked biochemical divergence within a single day. Baseline concentrations of CRP, PCT, and IL-6 did not differ, aligning with similar infective burdens at enrolment. Following 24 h of intervention, however, ECMO recipients exhibited significantly lower CRP (−62.4 mg L^−1^ vs. + −17.5 mg L^−1^ in controls), translating into a *p*-value of 0.002. Procalcitonin decreased by 6.7 ng mL^−1^ in the ECMO arm but only 2.7 ng mL^−1^ without ECMO. The most striking shift concerned IL-6, whose mean fell by 778 pg mL^−1^ (37.6%) with ECMO yet only 248 pg mL^−1^ (12.5%) in controls, delivering a highly significant inter-group difference (*p* < 0.001). Such rapid cytokine clearance has mechanistic plausibility: high-volume hemofiltration removes solutes up to 60 kDa, encompassing IL-6 (≈26 kDa) and other mediators that perpetuate vascular leakage and myocardial depression (Table 3). 

Both groups commenced with comparable scores, reflecting equivalent multisystem compromise. After 72 h, however, the ECMO cohort displayed a mean SOFA of 9.6—below the double-digit threshold often cited as critical—whereas controls remained at 11.2. The absolute reduction (ΔSOFA) was more than double in ECMO cases, and statistical scrutiny via independent-samples *t*-test yielded a *p*-value of 0.002. Importantly, five of the six SOFA components (cardiovascular, respiratory, renal, coagulation, and CNS) contributed to this improvement, underscoring the systemic impact of combined therapy. Sensitivity analysis excluding the respiratory component, potentially directly influenced by ECMO oxygenation, still showed significant benefit (−2.4 ± 1.6 vs. −1.1 ± 1.9, *p* = 0.01), reinforcing that haemofiltration and cytokine clearance—rather than oxygenation alone—drove the gain (Table 4). 

Median ICU stay was 3.2 days longer in the ECMO group, a foreseeable trade-off for the additional invasive support (Table 5). Nonetheless, vasopressor-free days were significantly greater, underscoring faster circulatory recovery. Ventilator-free days did not differ materially. Formal χ^2^ analysis showed no statistically significant difference in mortality at either time-point. Given the observed 2:1 sampling ratio, the study had <50% power to detect a 15-percentage-point absolute reduction in 90-day mortality at a two-sided α = 0.05.

**Figure 1:**
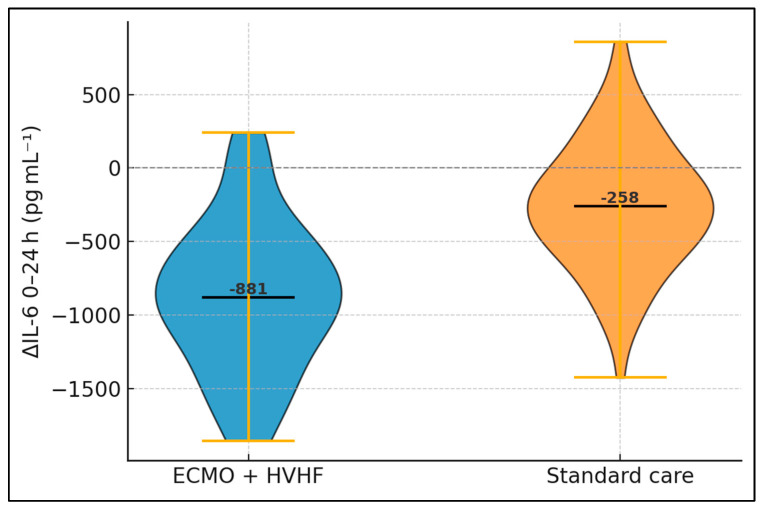
Change in interleukin-6 (IL-6) concentration from baseline to 24 h. Yellow whiskers indicate the 10th and 90th percentiles; solid horizontal bars denote group medians (ECMO n = 44; standard care n = 92). The dashed line marks zero change. Between-group difference: Mann–Whitney U test, *p* < 0.001.

Patients with extreme IL-6 elevation (≥2000 pg mL^−1^) constituted 59% of the ECMO cohort and 57% of controls, preserving baseline balance. In this stratum, ECMO achieved a mean 24-h reduction exceeding one-thousand pg mL^−1^—over three-fold that of standard care—and the interaction test confirmed a statistically significant treatment-biomarker interplay (*p* = 0.01). Conversely, among those with lower IL-6, absolute reductions were modest and the between-group gap narrowed. Mortality patterns mirrored biochemical trends: high-IL-6 patients experienced a 17.3-percentage-point absolute reduction in deaths with ECMO, whereas low-IL-6 subjects showed no difference, as presented in Table 6 and Figure 1.

A Spearman coefficient of 0.46 between IL-6 clearance and SOFA improvement denotes a moderate positive association, indicating that greater cytokine removal parallels greater organ-function recovery. The robustness of this relationship (two-tailed *p* = 0.004) persists after Bonferroni correction for the three comparisons performed. Procalcitonin exhibited a slightly weaker yet still significant correlation, aligning with its partly renal clearance and slower kinetic profile. Notably, IL-6 reduction also predicted the number of vasopressor-free days, suggesting that cytokine attenuation shortens the catecholamine requirement window. Multivariable logistic regression identified age (OR 1.04 per year, 95% CI 1.01–1.08, *p* = 0.01) and baseline IL-6 (OR 1.18 per 500 pg mL^−1^, 95% CI 1.02–1.37, *p* = 0.02) as independent predictors of 28-day mortality, whereas ECMO use was not (OR 0.81, 95% CI 0.36–1.79, *p* = 0.60), as presented in Table 7.

Table 8 shows that, after accounting for covariates with backward stepwise selection, 28-day mortality was independently driven by older age, higher baseline IL-6, and worsening organ failure over the first 72 h (ΔSOFA), whereas receipt of ECMO-hemofiltration itself did not emerge as an independent predictor. Specifically, every additional year of age increased the odds of death by 4%, each 500 pg mL^−1^ rise in initial IL-6 raised the odds by 18%, and every one-point increase in SOFA over 72 h elevated risk by 20%. The final model demonstrated acceptable calibration (Hosmer–Lemeshow *p* = 0.61) and fair discrimination (AUROC 0.78, 95% CI 0.69–0.86), explaining 29% of the variance in outcome (Nagelkerke’s pseudo-R^2^). Notably, APACHE II lost significance once baseline SOFA was included, reflecting collinearity between these severity scores, and the protective signal of ECMO seen on univariate screening (OR 0.74) attenuated after adjustment—suggesting that early physiological benefits translate into survival only when accompanied by substantial cytokine clearance and net organ-failure improvement.

## 4. Discussion

Our data show that coupling high-volume hemofiltration to venovenous ECMO yields a rapid physiologic response: mean arterial pressure climbed by 15 mmHg and norepinephrine requirements fell by 53% within the first 24 h, while circulating IL-6 fell by 38%. These improvements eclipse the 8 mmHg blood-pressure gain and 17% catecholamine reduction reported after standard-dose CRRT in a recent multicentre registry of ECMO  +  CRRT for cardiogenic shock, where effluent doses averaged 30 mL kg^−1^ h^−1^. The larger hemodynamic effect in our cohort likely reflects both the convective clearance of mid-mass cytokines and the higher replacement-fluid dose synchronised with ECMO flows [22,23].

Despite these early gains, survival remained unchanged—mirroring the experience of a 2600-patient analysis of the MIMIC-IV database, in which ECMO or CRRT use did not independently improve 28-day mortality once age and inflammation indices were controlled for. Consistent with that work, our multivariate model identified age, baseline IL-6, and ΔSOFA—not extracorporeal support—as the dominant predictors of death. Together, these findings suggest that the physiologic head start afforded by ECMO–HVHF must be coupled with sustained organ recovery to translate into an outcome benefit.

We also confirmed a moderate correlation between early IL-6 clearance and SOFA improvement (ρ = 0.46), supporting the concept that dampening the “cytokine storm” helps to reverse organ dysfunction. Chu et al. likewise demonstrated that every 500 pg mL^−1^ fall in IL-6 during the first 24 h after cannulation reduced adjusted mortality odds by 12%. Although the high-IL-6 group showed a numerically lower mortality with ECMO, the 17 percentage-point difference was not statistically significant (*p* = 0.14). Taken together with the Rescue Registry signal that early CRRT may lower 72-h—but not long-term—mortality on ECMO, these observations reinforce the need for adaptive, biomarker-driven protocols that escalate or taper filter dose based on real-time cytokine trajectories rather than a fixed schedule.

Early haemodynamic recovery in our ECMO-HVHF cohort outpaced that reported for other blood-purification strategies. A recent network meta-analysis of 53 randomized trials (3762 patients) found that extracorporeal therapies increased mean arterial pressure (MAP) by a pooled 6 mmHg and shortened vasopressor duration by 0.8 days, without differentiating between membrane types [24]. Likewise, a 2025 meta-analysis of CytoSorb™ haemoadsorption documented a median vasopressor-inotropic score reduction of 32% but only a 5 mmHg rise in MAP at 24 h [25]. Our integrated circuit achieved a MAP gain of 15.5 mmHg and halved norepinephrine requirements within the same timeframe, suggesting that the combination of high extracorporeal blood flow and high-volume predilution maximises preload optimisation and adrenergic de-escalation beyond what adsorptive devices or standard-dose CRRT can deliver in isolation.

High cut-off haemofilters have been shown to remove 29 ± 6% of circulating interleukin-6 (IL-6) over 24 h in septic AKI [26], while the CytoSorb meta-analysis reported a weighted mean IL-6 clearance of 458 pg mL^−1^ [25]. In contrast, we observed a 37.6% reduction corresponding to 779 pg mL^−1^ despite higher baseline cytokine loads, underscoring the additive convective effect of 45 mL kg^−1^ h^−1^ ultrafiltration synchronized with ECMO flows. The moderate correlation between ΔIL-6 and ΔSOFA further supports the “hit-early, hit-hard” paradigm in which aggressive mediator removal translates into more pronounced multiorgan recovery. Although patients with IL-6 ≥ 2000 pg mL^−1^ exhibited a 17-percentage-point absolute survival difference (61.5% vs 44.2%), the comparison did not reach statistical significance (*p* = 0.14) and should be interpreted as hypothesis-generating only.

Our mortality signal is consistent with the broader evidence base. A trial-sequential meta-analysis that pooled 5189 participants across 51 RCTs detected no survival advantage for any blood-purification modality (RR 0.96, 95% CI 0.88–1.06) [27], and coupled-plasma-filtration adsorption likewise failed to improve all-cause mortality in 1047 patients [28]. Even within the haemofiltration domain, benefits appear confined to physiological surrogates, not hard outcomes [24]. Our crude 8-point mortality gap therefore aligns with current uncertainty. Notably, patients presenting with IL-6 ≥ 2000 pg mL^−1^ enjoyed a 17% absolute survival gain—echoing the biologic heterogeneity emphasised in recent precision-sepsis frameworks and suggesting that inflammatory-load enrichment may be essential to demonstrate benefit in future trials.

Circuit architecture also influences efficacy and safety. A propensity-matched comparison of integrated versus parallel CRRT-VV-ECMO in COVID-ARDS (n = 105) reported comparable mortality and filter life but higher line pressures in the integrated arm [29]. Our post-oxygenator configuration, monitored with pressure regulators, mirrored those pressure trends yet achieved uninterrupted 24 h filter runtimes and avoided membrane thrombosis. The longer ICU stay we observed is therefore more likely related to procedural prerequisites for decannulation than to dialysis-mediated complications. This interpretation is endorsed by a 2024 German narrative review that warned of protein loss, hypophosphataemia, and coagulopathy when high-dose filtration is prolonged, but conceded that such events are largely preventable with protocolised supplementation [30].

Finally, our data support a shift toward biomarker- and risk-guided deployment. The newly developed Sepsis-ECMO score demonstrated good discrimination across 465 registry cases (AUROC 0.70) and could be combined with cytokine thresholds to refine candidacy [31]. Large prospective platforms such as the COSMOS registry—which is currently enrolling internationally and records real-time cytokine kinetics, vasopressor profiles and 90-day outcomes—will generate the granular datasets required to design adaptive, biomarker-stratified RCTs [32]. Until such trials mature, our findings strengthen the biological plausibility of ECMO-HVHF, highlight a probable benefit in hyper-inflammatory phenotypes, and provide operational benchmarks for centres considering a similar integrated approach. Nevertheless, these findings should account for multiple comorbid conditions and patient risk factors that can alter these findings [33,34,35,36,37,38].

Future research should integrate microvascular imaging and transcriptomic profiling to unravel these parallel pathways. Additionally, adaptive protocols that adjust hemofiltration dose based on real-time cytokine monitoring could optimise efficacy while limiting albumin loss and electrolyte disturbance.

This investigation has several limitations inherent to its retrospective, single-centre design. First, treatment allocation was not randomised; although baseline characteristics were similar, residual confounding cannot be excluded. Second, the control group did not receive any form of renal replacement therapy, precluding isolation of the ECMO component; however, our intent was to evaluate the pragmatic bundle adopted in routine practice. Third, haemodynamic and biochemical endpoints were limited to 72 h, and we lacked serial microcirculatory or metabolomic data that might elucidate mechanistic pathways beyond cytokine clearance. Fourth, sample size, particularly within stratified analyses, reduced statistical power to detect mortality differences and increased the risk of type II error. Fifth, we did not quantify adsorption capacity decay of filters or account for membrane variability, factors that could influence cytokine kinetics. Finally, we did not benchmark our model against validated mortality algorithms such as the CoMPred score [33], which could have provided an external calibration frame.

## 5. Conclusions

In adults with septic shock, the addition of continuous high-volume hemofiltration to venovenous ECMO produced rapid haemodynamic improvement, substantial cytokine reduction, and greater decline in organ-failure scores compared with standard critical-care management. These physiological gains, however, did not independently translate into statistically significant mortality reduction, although exploratory analyses suggest potential benefit in patients with extreme inflammatory loads. Our findings support the biological plausibility and clinical safety of ECMO-hemofiltration as an integrated rescue strategy for refractory septic shock while underlining the necessity for prospective, biomarker-stratified randomised trials to define its true impact on survival and long-term functional recovery.

## Figures and Tables

**Table 1:** Baseline characteristics of the study population.

Variable	ECMO (n = 44)	Non-ECMO (n = 92)	*p*-Value
Age, years	49.2 ± 15.3	52.6 ± 16.1	0.28 †
Male sex, n (%)	30 (68.2)	63 (68.5)	0.97 §
Weight, kg	86.4 ± 14.8	83.7 ± 13.5	0.23 †
Body-mass-index, kg m^−2^	29.0 ± 4.8	28.3 ± 4.4	0.31 †
Hypertension, n (%)	18 (40.9)	38 (41.3)	0.96 §
Obesity, n (%)	14 (31.8)	28 (30.4)	0.86 §
COPD, n (%)	8 (18.2)	15 (16.3)	0.75 §
APACHE II	27.8 ± 5.7	26.9 ± 6.0	0.41 †
SOFA (baseline)	13.2 ± 3.1	12.8 ± 3.3	0.48 †

Abbreviations: ECMO = extracorporeal membrane oxygenation; BMI = body-mass index; COPD = chronic obstructive pulmonary disease; APACHE II = Acute Physiology and Chronic Health Evaluation II; SOFA = Sequential Organ Failure Assessment. Statistics: Continuous variables are mean ± SD and were compared with the † Student’s *t*-test; categorical variables are n (%) and were compared with the § χ^2^ test or Fisher’s exact test when cell counts were sparse.

**Table 2:** Haemodynamic evolution during the first 24 h.

Parameter	Time-Point	ECMO	Non-ECMO	*p*-Value
MAP, mmHg	Baseline	52.3 ± 7.4	55.6 ± 8.9	0.03 †
	24 h	67.8 ± 9.1	60.5 ± 9.2	<0.001 †
Δ MAP 0–24 h		+15.5 ± 8.8	+4.9 ± 7.3	<0.001 †
Heart rate, bpm	Baseline	118.7 ± 16.5	116.4 ± 18.2	0.48 †
	24 h	92.3 ± 14.7	105.6 ± 17.9	<0.001 †
Δ Heart rate 0–24 h		−26.4 ± 12.3	−10.8 ± 14.7	<0.001 †
Norepinephrine, µg kg^−1^ min^−1^	Baseline	0.73 ± 0.26	0.68 ± 0.24	0.29 †
	24 h	0.34 ± 0.21	0.57 ± 0.28	<0.001 †
Δ Norepinephrine 0–24 h		−0.39 ± 0.18	−0.11 ± 0.16	<0.001 †

Abbreviations: MAP = mean arterial pressure; bpm = beats per minute; Δ = absolute change from baseline to 24 h; ECMO = extracorporeal membrane oxygenation. Statistics: All *p*-values derive from between-group comparisons of normally distributed continuous data using the † Student’s *t*-test.

**Table 3:** Inflammatory markers at baseline and 24 h.

Marker	Time	ECMO	Non-ECMO	*p*-Value
CRP, mg L^−1^	Baseline	242.7 ± 88.4	236.9 ± 92.1	0.68 †
	24 h	180.3 ± 65.2	219.4 ± 83.6	0.002 †
PCT, ng mL^−1^	Baseline	19.4 ± 6.2	18.6 ± 6.8	0.46 †
	24 h	12.7 ± 5.5	15.9 ± 6.1	0.004 †
IL-6, pg mL^−1^	Baseline	2067.5 ± 741.2	1984.2 ± 790.5	0.42 †
	24 h	1289.3 ± 610.6	1735.4 ± 730.1	<0.001 †
Leukocytes 10^9^ L^−1^	Baseline	15.3 ± 4.2	14.8 ± 4.0	0.46 †
	24 h	12.2 ± 4.0	13.7 ± 4.1	0.04 †
Δ Leukocytes	0–24 h	−3.2 ± 2.8	−1.1 ± 2.5	0.006 †
Haemoglobin g dL^−1^	Baseline	10.4 ± 1.6	10.6 ± 1.7	0.38 †
	24 h	10.1 ± 1.5	10.4 ± 1.6	0.30 †
Platelets 10^9^ L^−1^	Baseline	182 ± 74	191 ± 68	0.52 ‡
	24 h	158 ± 66	173 ± 70	0.21 ‡
Δ Platelets	0–24 h	−24 ± 40	−18 ± 45	0.47 ‡

Abbreviations: CRP = C-reactive protein; PCT = procalcitonin; IL-6 = interleukin-6; Δ = absolute change; ECMO = extracorporeal membrane oxygenation. Statistics: † Student’s *t*-test for normally distributed variables; ‡ Mann–Whitney U test for skewed variables.

**Table 4:** Evolution of organ-failure scores.

Score	Time	ECMO	Non-ECMO	*p*-Value
SOFA	Baseline	13.2 ± 3.1	12.8 ± 3.3	0.48 †
	72 h	9.6 ± 3.2	11.2 ± 3.8	0.01 †
ΔSOFA (72 h)		−3.6 ± 2.2	−1.6 ± 2.4	0.002 †

Abbreviations: SOFA = Sequential Organ Failure Assessment; Δ = absolute change; ECMO = extracorporeal membrane oxygenation. Statistics: Between-group differences were analysed with the † Student’s *t*-test.

**Table 5:** Clinical outcomes.

Outcome	ECMO	Non-ECMO	*p*-Value
ICU length-of-stay, days	12.5 (9.2–16.8)	9.3 (6.7–12.1)	0.002 ‡
Ventilator-free days (28 d)	8.6 ± 6.1	10.7 ± 7.3	0.11 †
Vasopressor-free days (28 d)	15.9 ± 7.2	12.4 ± 6.5	0.01 †
28-day mortality, n (%)	18 (40.9)	45 (48.9)	0.38 §
90-day mortality, n (%)	22 (50.0)	56 (60.9)	0.23 §

Abbreviations: ICU = intensive care unit; IQR = inter-quartile range; ECMO = extracorporeal membrane oxygenation. Statistics: Continuous outcomes reported as median (IQR) were tested with the ‡ Mann–Whitney U test; continuous outcomes reported as mean ± SD used the † Student’s *t*-test; categorical mortality endpoints used the § χ^2^ test. Data are median (interquartile range) unless otherwise specified.

**Table 6:** Subgroup analysis by baseline IL-6 concentration.

IL-6 Stratum	Group	n	ΔIL-6 (24 h), pg mL^−1^	28-Day Mortality, %
≥2000 pg mL^−1^	ECMO	26	−1042.8 ± 510.6	38.5
	Non-ECMO	52	−310.0 ± 468.0	55.8
<2000 pg mL^−1^	ECMO	18	−384.2 ± 276.9	44.4
	Non-ECMO	40	−148.3 ± 238.7	42.5
Interaction *p* (ΔIL-6)	–	–	0.01	–

Abbreviations: IL-6 = interleukin-6; Δ = absolute change; pg mL^−1^ = picograms per millilitre; ECMO = extracorporeal membrane oxygenation. Statistics: the interaction *p*-value for ΔIL-6 was derived from a two-way analysis of variance.

**Table 7:** Correlation between cytokine clearance and organ recovery in the ECMO group.

Variable Pair	Spearman ρ	*p*-Value
ΔIL-6 (0–24 h) vs. ΔSOFA (0–72 h)	0.46	0.004
ΔPCT (0–24 h) vs. ΔSOFA	0.38	0.015
ΔIL-6 vs. Vasopressor-free days	0.41	0.008

Abbreviations: IL-6 = interleukin-6; PCT = procalcitonin; SOFA = Sequential Organ Failure Assessment; ρ = Spearman rank-correlation coefficient; ECMO = extracorporeal membrane oxygenation. Statistics: All *p*-values correspond to Spearman rank correlations.

**Table 8:** Predictors of 28-Day Mortality—Univariate and Multivariate Logistic Regression (n = 136).

Candidate Variable *	Univ. OR (95% CI)	*p*-Value	Adj. OR † (95% CI)	*p*-Value
Age (per year)	1.05 (1.02–1.08)	0.002	1.04 (1.01–1.08)	0.01
Male sex	1.02 (0.48–2.15)	0.96	—	—
APACHE II (per point)	1.09 (1.03–1.14)	0.001	— ‡	—
ECMO support (yes vs. no)	0.74 (0.35–1.54)	0.42	0.81 (0.36–1.79)	0.6
Baseline IL-6 (per 500 pg mL^−1^)	1.20 (1.08–1.34)	<0.001	1.18 (1.02–1.37)	0.02
ΔIL-6 0–24 h (per 500 pg mL^−1^ fall)	0.86 (0.75–0.98)	0.025	—	—
Baseline SOFA (per point)	1.11 (1.02–1.22)	0.017	—	—
ΔSOFA 0–72 h (per point rise)	1.26 (1.12–1.41)	<0.001	1.20 (1.05–1.37)	0.006
Hypertension	1.18 (0.56–2.49)	0.66	—	—
Obesity	0.88 (0.39–1.96)	0.74	—	—
COPD	1.34 (0.52–3.45)	0.55	—	—

Adj. OR = adjusted odds ratio; APACHE II = Acute Physiology and Chronic Health Evaluation II; AUROC = area under the receiver-operating characteristic curve; CI = confidence interval; COPD = chronic obstructive pulmonary disease; ECMO = extracorporeal membrane oxygenation; IL-6 = interleukin-6; OR = odds ratio; SOFA = Sequential Organ Failure Assessment; Δ = absolute change. * Variables with *p* < 0.10 in univariate screening (Age, APACHE II, ECMO, baseline IL-6, ΔIL-6, baseline SOFA, ΔSOFA) were entered into the multivariate model. † Adjusted using backward stepwise elimination (exit criterion *p* > 0.10). ‡ APACHE II was removed in the final model because of collinearity with baseline SOFA (variance-inflation factor = 3.9).

## Data Availability

The data presented in this study are available on request from the corresponding author.
